# Bacterial Species Associated With Human Inflammatory Bowel Disease and Their Pathogenic Mechanisms

**DOI:** 10.3389/fmicb.2022.801892

**Published:** 2022-02-24

**Authors:** Li Zhang, Fang Liu, Jessica Xue, Seul A. Lee, Lu Liu, Stephen M. Riordan

**Affiliations:** ^1^School of Biotechnology and Biomolecular Sciences, University of New South Wales, Sydney, NSW, Australia; ^2^Faculty of Medicine, Monash University, Melbourne, VIC, Australia; ^3^School of Medical Sciences, University of New South Wales, Sydney, NSW, Australia; ^4^Gastrointestinal and Liver Unit, Prince of Wales Hospital, University of New South Wales, Sydney, NSW, Australia

**Keywords:** inflammatory bowel disease, chronic inflammation, *Campylobacter concisus*, adherent-invasive *Escherichia coli*, *Fusobacterium nucleatum*, *Mycobacterium avium* subspecies *paratuberculosis*, *Fusobacterium varium*

## Abstract

Inflammatory bowel disease (IBD) is a chronic inflammatory condition of the gastrointestinal tract with unknown etiology. The pathogenesis of IBD results from immune responses to microbes in the gastrointestinal tract. Various bacterial species that are associated with human IBD have been identified. However, the microbes that trigger the development of human IBD are still not clear. Here we review bacterial species that are associated with human IBD and their pathogenic mechanisms to provide an updated broad understanding of this research field. IBD is an inflammatory syndrome rather than a single disease. We propose a three-stage pathogenesis model to illustrate the roles of different IBD-associated bacterial species and gut commensal bacteria in the development of human IBD. Finally, we recommend microbe-targeted therapeutic strategies based on the three-stage pathogenesis model.

## Introduction

Inflammatory bowel disease (IBD) is a chronic inflammatory condition of the gastrointestinal tract (Ng et al., 2017). Crohn’s disease (CD) and ulcerative colitis (UC) are the two major clinical forms of IBD. IBD can occur in all age groups but most often in late adolescents and young adults (Johnston and Logan, 2008). The etiology of IBD is not completely understood. Studies suggest that environmental factors, gut microbes and genetics play a role in the development of IBD (Sairenji et al., 2017). The pathogenesis of IBD results from immune responses to microbes in the gastrointestinal tract, demonstrated by studies in patients with IBD and using animal models (De Souza and Fiocchi, 2016). The human gastrointestinal tract is colonized by millions of bacterial cells consisting of more than 500 bacterial species (Thursby and Juge, 2017). These microbes maintain a homeostatic relationship with the mucosal immune system in healthy individuals, thus referred to as commensal bacteria. The microbes that trigger the breakdown of the homeostasis between the mucosal immune system and the commensal microbes of the gut microbiota in patients with IBD are still not clear after decades of research. In this article, we review bacterial species that are associated with human IBD and their pathogenic mechanisms. We also propose a three-stage pathogenesis model to illustrate the roles of IBD-associated bacterial species and gut commensal bacteria in the development of human IBD. Finally, we recommend microbe-targeted therapeutic strategies based on the proposed three-stage pathogenesis model.

## Interactions Between Gut Microbiota and the Mucosal Immune System in Homeostasis

There are dynamic interactions between gut microbes and the mucosal immune system in healthy individuals, however, such interactions do not induce inflammation. In order to better explain the roles of different bacterial species in the pathogenesis of IBD, we firstly summarize the main strategies that maintain the homeostasis between gut microbiota and the mucosal immune system in healthy conditions.

The first strategy is to limit the contact of luminal microbes with the host cells and tissues. This is achieved via the barrier formed by mucus, antimicrobial molecules secreted by intestinal epithelial cells, secretary IgA produced by plasma cells in the lamina propria and the intestinal epithelium (Bankaitis et al., 2018). The mucus that covers the small intestine is a loose and unattached layer with high concentrations of antibacterial molecules which limits the numbers of bacteria that can reach the epithelium and Peyer’s patches (Johansson et al., 2013; Sheng et al., 2013). The mucus that covers the large intestine has two layers. The inner mucus layer is firmly attached to intestinal epithelial cells and free of bacteria in healthy individuals. The outer mucus layer is loose and has bacterial colonization (Johansson et al., 2013). The intestinal epithelium contains four major types of cells that are derived from stem cells located in the crypts (Bankaitis et al., 2018). Most of the intestinal epithelial cells are enterocytes which are absorptive cells. The others are secretary cells including goblet cells, Paneth cells and enteroendocrine cells. Goblet cells secrete mucus. The epithelial cells are connected by tight junction proteins.

The second strategy is the strategically arranged innate pattern recognition receptors (PRRs) in the intestinal epithelium. Intestinal epithelial cells express many different types of PRRs, located on the surface or inside of cells. These receptors recognize and respond to intruding pathogens but maintain a tolerance to gut microbiota and food components when under a homeostatic state. Such a tolerance is maintained by the low expression of these receptors and their spatial location in healthy conditions (Santaolalla and Abreu, 2012). For example, Toll-like receptor-4 (TLR4) and MD-2 are expressed at a low level in normal intestinal epithelial cells and they do not respond to lipopolysaccharide (LPS), a major component of Gram-negative bacteria (Abreu et al., 2002). Furthermore, TLRs have a polarized distribution; they are located at the basolateral surfaces instead of apical surface, which further limits the response of the intestinal epithelium to products of commensal gut microbes that have crossed the mucus layer (Abreu et al., 2002; Burgueño and Abreu, 2020).

The third strategy is the unique regulatory network of the mucosal immune system. Macrophages in the lamina propria of the intestinal mucosa play an important role in both maintenance of intestinal homeostasis and defense of pathogens. Under homeostatic conditions, lamina propria macrophages uptake luminal bacteria or their products. These macrophages produce little or no pro-inflammatory cytokines after taking luminal antigens (Denning et al., 2007; Smith et al., 2011). Instead, they produce anti-inflammatory molecules such as interleukin (IL)-10 (Denning et al., 2007). Furthermore, CD11b^+^ F4/80^+^ CD11c^–^macrophages and CD103^+^ dendritic cells induce antigen-specific regulatory T cells (Treg) in the mesenteric lymph nodes (Denning et al., 2007; Bain and Schridde, 2018). The intestinal tissues contain high numbers of Forkhead box P3 (Foxp3) Treg cells that are important in preventing intestinal inflammation. Foxp3^+^ Treg cells control host immune responses through multiple mechanisms such as the production of immune suppression cytokines of tumor growth factor (TGF)-β and IL-10, changing antigen presenting cell functions such as down regulation of B7 molecules on dendritic cells (Harrison and Powrie, 2013).

Gut microbiota contributes to the development and function of the immune system. Animals raised in germ-free conditions where no live microorganisms were present had fewer and smaller Peyer’s patches and mesenteric lymph modes, reduced numbers of CD4^+^ T cells and intraepithelial CD8^+^ T cells in the lamina propria, and reduced numbers of IgA producing B cells and plasma cells (Round and Mazmanian, 2009). Isolated lymphoid follicles in the intestinal tract are formed and matured after birth, which are impaired as well in germ-free mice (Bouskra et al., 2008). Animals raised under germ-free conditions are less resistant to pathogen infections. Guinea pigs raised under conventional conditions resisted oral challenge with *Shigella flexneri*. However, germ-free guinea pigs receiving a similar challenge died (Sprinz et al., 1961). Germ-free mice also showed impaired abilities in eliminating *Listeria monocytogenes* after intraperitoneal infection and this may be due to lack of L-selectin ^+^CD44^+^ T cells in sites of inflammation (Inagaki et al., 1996). These findings show that the products of gut microbiota continuously stimulate the mucosal immune system in homeostatic states, which serves as an important training strategy for host protection.

## Bacterial Species Associated With Human Inflammatory Bowel Disease

Studies have searched for bacterial species that may play a role in the development of IBD. Here, we will review individual bacterial species that were found to be associated with human IBD according to the chronological order of the discovery year of their association with IBD. IBD-associated bacterial species with pathogenic mechanisms studied are listed in Table 1.

**TABLE 1:** Bacterial species that are associated with human IBD and their pathogenic mechanisms.

Bacterial species	Detection methods	Pathogenic mechanisms
*Clostridium difficile*	Bacterial isolation	• Inactivating Rho and Ras GTPase by glucosylation.
	PCR detection of toxins	• Increasing epithelial permeability and luminal fluid accumulation
		• Causing intestinal epithelial cell death
		• Inducing the production of IL-8, TNF-α, IL-1 and IL-6
*Mycobacterium avium* subspecies	Bacterial isolation	• Binding to M cells through fibronectin.
*paratuberculosis* (MAP)	PCR detection of IS900	• Survival and proliferation within macrophages.
		• Mycobacterial products can scavenge reactive oxygen intermediates.
Enterotoxigenic *B. fragilis*	PCR detection of *bft* gene	• Caused the loss of E-cadherin in intestinal epithelial cells.
	Bacterial isolation	• Induced production of IL-8 production in intestinal epithelial cells.
		• Induced colitis in mice models and promoted intestinal inflammation via Th17 response.
*Fusobacterium varium*	Bacterial isolation Western blot ELISA	• Produced a high level of *n*-butyric acid in the bacterial culture supernatants which killed Vero cells.
		• *F. varium* culture supernatants induced colonic ulceration, crypt abscesses, inflammatory cell infiltration and apoptotic changes in mice.
		• *F. varium* induced adaptive immune response, antibodies to *F. varium* were detected in patients with UC.
Adherent-invasive *Escherichia coli* (AIEC)	Bacterial isolation Adhesion and invasion assay	• AIEC strains adhere to and invade intestinal epithelial cells via interactions of the common type 1 pili adhesion FimH and CEACAM6. AIEC strains interact with M cells on the surface of Peyer’s patches, which facilitated the translocation of AIEC across monolayers of M cells.
		• AIEC strains induced production of IL-8 in epithelial cells.
		• AIEC strains resist the defense from macrophages by avoidance of autophagy.
		• The persistence of AIEC in macrophages induced increased production of TNF-α and IL-6. AIEC strain LF82 induced intestinal inflammation in transgenic mice that express human CEACAMS and in conventional mice treated with streptomycin.
*Campylobacter concisus*	PCR targeting 16S rRNA gene	• Caused intestinal epithelial cell death.
	Sequencing PCR products	• Affected tight junction proteins.
	Bacterial isolation	• *C. concisus* increased TLR4, MD-2 and COX-2 in intestinal epithelial cells.
		• Induced IL-8 production in intestinal epithelial cells and macrophages.
		• *C. concisus* Zot protein caused intestinal epithelial cell death and enhanced the responses of macrophages to commensal *E. coli.*
*Fusobacterium nucleatum*	Bacterial isolation	• Caused intestinal epithelial death via activation of autophagy.
	Invasion assay	• Affected the expression and distribution of zonula occludens-1 (ZO-1) and occludens.
		• Aggravated colitis via skewing proinflammatory M1 macrophage.
		• Induced Th1 and Th17 subset T cell differentiations, promoted the secretion of proinflammatory cytokines such as TNF-α, IL-1β, IL-6, and IL-17.

*Individual bacterial species with known pathogenic mechanisms are listed in this table according to the chronological order of the discovery year of their association with IBD. C. difficile is mainly associated with relapsed IBD but was also detected in newly diagnosed IBD. The studies of other bacterial species in the table were mainly in newly diagnosed IBD patients. For gut microbiota studies, please refer to the summary in the text.*

### *Clostridium difficile* and Other Enteric Pathogen Infections in Inflammatory Bowel Disease

*C. difficile* is a Gram-positive spore-forming anaerobic bacterium, which is ubiquitous in nature and also colonizes the human intestinal tract (Ryan and Ray, 2004). *C. difficile* causes diarrhea and colitis, often in individuals who have been treated with antibiotics for other medical conditions.

The implications of *C. difficile* infection in the therapies for IBD relapses were suggested in 1980 (Lamont and Trnka, 1980). Later studies suggest that *C. difficile* may contribute to the relapse or trigger the development of IBD in some patients. Patients with IBD are at a higher risk of developing *C. difficile* infection. Studies of hospitalized patients from United States found that *C. difficile* was more common in patients with UC (3.9%) as compared to non-IBD patients (1.2%), and an adjusted odds ratio for *C. difficile* infection in patients with IBD, CD and UC were 2.9, 2.1, and 4.0, respectively (Meyer et al., 2004; Rodemann et al., 2007). Further studies from United States showed that enteric pathogens were detected in up to 20% of relapsed IBD patients with *C. difficile* being the most common pathogen (up to 90%) of the detected pathogens (Binion, 2012; Pant et al., 2013). IBD patients with *C. difficile* infection have worse outcomes and experience higher rates of recurrence (Issa et al., 2007; Jodorkovsky et al., 2010; Navaneethan et al., 2012). While these findings support a role of *C. difficile* infection in participating the pathogenesis in relapsed IBD, 10% of *C. difficile* infection occurred at the time of IBD diagnosis. This and the finding that IBD patients in remission had a significantly higher presence of toxigenic *C. difficile* in their intestinal tract as compared to healthy controls (8.2 vs. 1%) suggest that *C. difficile* infection may be a risk factor for the development of IBD (Clayton et al., 2009). A more recent study from the Netherlands employing both retrospective and prospective approaches found a low prevalence of *C. difficile* that was not associated with IBD. Their finding indicated that *C. difficile* is not a common trigger for exacerbations of IBD in clinical practice in the Netherlands (Masclee et al., 2013).

The major virulence factors of *C. difficile* are the two toxins encoded by *tcdA* and *tcdB* genes (McDonald et al., 2005; Belyi and Aktories, 2010; Aktories et al., 2017). These toxins inactivate host Rho and Ras family GTPase by glucosylation leading to the disruption of epithelial cytoskeleton and tight junctions, which may contribute to the increased epithelial permeability and luminal fluid accumulation associated with *C. difficile* infection (Kim et al., 2005). TcdA and TcdB toxins also cause cell death via caspase-dependent and caspase-independent apoptosis, further damaging the intestinal epithelial barrier (Hippenstiel et al., 2002; Qa’Dan et al., 2002; Matarrese et al., 2007; Nottrott et al., 2007). Both toxins also induce the production of proinflammatory cytokines in intestinal epithelial and immune cells, including IL-8, TNF-α, IL-1, and IL-6 (Savidge et al., 2003). By using multiomic analysis, it was found that the most discriminating metabolite between IBD and IBD infected with *C. difficile* was isocaproyltaurine, a covalent conjugate of a *C. difficile* fermentation product isocaproate and taurine from damaged tissue (Bushman et al., 2020).

In addition to *C. difficile*, other enteric pathogens were also detected in patients with flares of IBD. A recent study from United States found that compared to patients without IBD, patients with CD had a higher prevalence of norovirus (*p* = 0.05) and *Campylobacter* (*p* = 0.043). Compared to patients without IBD, patients with UC had a lower prevalence of norovirus (*p* = 0.049) and a higher prevalence of *Campylobacter* (*p* = 0.013), *Plesiomonas* (*p* = 0.049) and *Escherichia coli* (*p* < 0.001). However, these enteric infections did not impact IBD outcomes (Axelrad et al., 2018). A recent study from UK also reported that patients with IBD in primary care were more likely to present with common infections including gastrointestinal infections of *C. difficile*, *Salmonella*, *Shigella* and *Campylobacter* (Irving et al., 2021). *Campylobacter* species isolated in diagnostic laboratories are mainly *Campylobacter jejuni*.

### *Mycobacterium avium* Subspecies *paratuberculosis*

*M. avium* is a mycobacterial species that is commonly present in the environment, which consists of four subspecies, including *M. avium* subspecies *avium*, *M. avium* subspecies *paratuberculosis* (MAP), *M. avium* subspecies *hominissuis* and *M. avium* subspecies *silvaticum* (Uchiya et al., 2017). MAP is present in about 70% of dairy herds without causing problems (Nielsen et al., 2000). However, it can cause Johne’s disease (chronic intestinal inflammation in distal small intestine) in cattle (Singh et al., 2014). Due to the similarities in histopathology between Johne’s disease and CD, it has been proposed that MAP may play a role in CD. In 1984, an unclassified mycobacterial species was isolated from gastrointestinal tissues of two patients with CD (Chiodini et al., 1984). A 7-day-old goat inoculated orally with 50 mg of the isolated organism developed a granulomatous lesion at the distal small intestine. The authors of this study concluded that their findings raise the possibility that a *Mycobacterium* plays an etiologic role in at least some cases of CD (Chiodini et al., 1984). Following this, multiple studies examined the association between MAP and CD, using bacterial cultivation or a PCR method targeting the insertion element (IS 900) in the genome of MAP. These studies generated controversial results and the details of these studies were previously reviewed by Naser et al. (2014).

Several clinical studies using anti-mycobacterial antibiotics in the treatment of CD reported beneficial effects. These studies used a combination of multiple antibiotics including rifabutin, clofazimine and clarithromycin, plus either ciprofloxacin, metronidazole, or ethambutol (Agrawal et al., 2020a,b). However, these studies did not identify whether patients included in the treatment were positive for MAP. Furthermore, the multiple antibiotics used in these studies would have had effects on other non-mycobacterial bacterial species. Although these studies did not prove MAP causes CD, they provided evidence supporting that bacterial species are involved in the pathogenesis of CD. Other studies also reported that different antibiotics such as nitroimidazoles and clofazimine also appear to be effective in the treatment of CD, further supporting that bacterial species are involved in the pathogenesis of CD (Feller et al., 2010).

The pathogenic mechanisms of MAP in Johne’s disease were studied. MAP is taken in by microfold (M) cells following recognition of the bacterium by the fibronectin receptor (Secott et al., 2004). MAP bacteria that travel across the intestinal epithelium are phagocytosed by macrophages via complement receptors. Infected macrophages form granulomas and become a latent infection or active disease (Birkness et al., 2007; Rice et al., 2019).

### Enterotoxigenic *Bacteroides fragilis*

*B. fragilis* is a Gram-negative anaerobe, a commensal bacterium of the intestinal tract. However, a particular strain, namely enterotoxigenic *B. fragilis* strain, which produces an enterotoxin encoded by the *bft* gene, was reported to be associated with IBD disease activation or flare-up in year 2000 by a study from United States (Prindiville et al., 2000). The enterotoxin encoded by the *bft* gene is a secreted metalloprotease (Moncrief et al., 1995; Franco et al., 1997). A study in 2017 from Iran reported that *bft* gene was detected in 51.4% of intestinal biopsies from patients with UC and 1.6% non-IBD samples (Zamani et al., 2017). The *B. fragilis* enterotoxin cleaved the adherens junction protein E-cadherin and induced IL-8 production in intestinal epithelial cells (Wu et al., 1998, 2004; Kim et al., 2001). The enterotoxigenic *B. fragilis* strain induced persistent colitis with inflammatory cell infiltration, crypt abscesses and epithelial ulceration in wild type specific-pathogen free C57BL/6 mice and lethal colitis in germ-free C57BL/6 mice (Rhee et al., 2009). In multiple intestinal neoplasia mice, enterotoxigenic *B. fragilis* induced colitis via T17 response pathway and also induced colonic tumors (Wu et al., 2009). A recent study from the Netherlands reported a different result. They detected a higher prevalence of *B. fragilis* in patients with active CD as compared to patients in remission, however, *bft*-positive *B. fragilis* was not higher and they also found that *bft*-positive *B. fragilis* increased epithelial resistance rather than damaging the barrier (Becker et al., 2021).

### Fusobacterium varium

*F. varium* is a Gram-negative bacterium. It was reported in 2002 from Japan that *F. varium* in colonic mucosa of patients with UC was significantly higher than that in CD and healthy controls detected using immunohistochemical method (Ohkusa et al., 2002). This study also detected a significantly higher antibodies to extracts of *F. varium* antigens (Ohkusa et al., 2002). In a following study, the same research group examined the cytotoxicity of supernatants of 20 bacterial species isolated from intestinal tissues of patients with UC. Only the supernatant of *F. varium*, which was found to contain high concentration of butyric acid, killed Vero cells (Ohkusa et al., 2003). Enemas containing *F. varium* culture supernatants given to mice induced colonic ulceration, crypt abscesses, inflammatory cell infiltration and apoptotic changes (Ohkusa et al., 2003). In a different study, Tahara et al. (2015) detected *Fusobacterium* in inflamed intestinal tissues of 152 Japanese UC patients using quantitative real-time PCR and found that *Fusobacterium* species were commonly present in patients with UC and was associated with mild clinical phenotypes of UC. However, the PCR primers that Tahara et al. (2015) used were not specific for *F. varium*.

A recent study in 2016 from Korea isolated *Fusobacterium* species from colonic biopsies of 51 (15.6%) patients with IBD (26 CD and 25 UC). *Fusobacterium* bacteria were isolated from eight of the 51 patients with IBD, however, only two were *F. avium* (Lee et al., 2016).

### Adherent-Invasive *Escherichia coli*

*E. coli* is a Gram-negative facultative bacterium, which is a commensal bacterium of the human gut. However, some strains of *E. coli* are human enteric pathogens. The adherent-invasive *Escherichia coli* (AIEC) was reported to be associated with ileal CD in the adult population (Darfeuille-Michaud et al., 2004; Martin et al., 2004; Sasaki et al., 2007). AIEC strains were defined based on their ability to adhere to and invade intestinal epithelial cell lines assessed (Darfeuille-Michaud et al., 2004). Various cell lines such as Caco-2, intestine-407 and HT-29 cells were used in assessing AIEC strains. AIEC strains do not have specific virulence factors. Comparative genomic analysis of CD associated AIEC strains and non-AIEC strains did not identify any molecular marker that can differentiate AIEC strains from non-AIEC strains (O’Brien et al., 2017). In the past 20 years, extensive studies have been conducted to examine the pathogenic mechanisms of AIEC strains.

AIEC strains adhere to and invade intestinal epithelial cells. AIEC adhesion to intestinal epithelial cells was via interactions of the common type 1 pili adhesion FimH and carcinoembryonic antigen cell adhesion molecule 6 (CEACAM6) (Boudeau et al., 2001; Barnich et al., 2007; Carvalho et al., 2009; Dreux et al., 2013). CEACAM6 is a glycoprotein that is abnormally expressed in intestinal epithelial cells of patients with ileal CD (Barnich et al., 2007). AIEC strains also used long polar fimbriae to interact with M cells on the surface of Peyer’s patches, which facilitated the translocation of AIEC across monolayers of M cells (Chassaing et al., 2011).

AIEC strains resist the defense from macrophages. Macrophages play a critical role in clearance of bacterial infection. In macrophages, while AIEC bacteria targeted early by the autophagic machinery were degraded, those that escaped early uptake by autophagy were able to survive and replicate inside macrophages (Lapaquette et al., 2012). Impaired ATG16L1, IRGM, or NOD2, which are molecules affecting autophagy or bacterial sensing, favored AIEC persistence in macrophages and induced increased production of tumor necrosis factor (TNF)-α and IL-6 (Lapaquette et al., 2012). AIEC induced granulomas *in vitro* (Lapaquette et al., 2012). AIEC strains promote inflammation. AIEC strains induced production of cytokines such as IL-8, TNF-α, and IL-6 in both epithelial cells and macrophages (Subramanian et al., 2008; Lapaquette et al., 2012). Survival and replication of AIEC in macrophages did not cause macrophage death, but increased production of TNF-α and IL-6 (Lapaquette et al., 2012). AIEC strain LF82 induced intestinal inflammation in CEABAC10 mice, transgenic mice that express human CEACAMS (Carvalho et al., 2009). In conventional mice treated with streptomycin, AIEC strains also induced chronic intestinal inflammation and intestinal fibrosis (Small et al., 2013). CD8^+^ T cells showed a protective role in AIEC infection in streptomycin-treated mice, and depletion of CD8^+^ T cells increased cecal AIEC load, intestinal pathology, and fibrosis in C56BL/6 mice (Small et al., 2013).

### Enteric *Helicobacter* Species

*Helicobacters* are Gram-negative bacterial species. *H. pylori* is the well-known human gastric pathogen. Some *Helicobacter* species were also isolated from intestinal tract of humans and animals, which are referred to as enteric *Helicobacter* species. By using a genus PCR, a study from Germany in 2004 detected a higher presence of *Helicobacter* species in colonic and ileal biopsies of patients with CD (12%), UC (17%) than controls (4%), however the difference did not reach statistical significance (Bohr et al., 2004). Sequencing of the genus PCR products showed that the detected *Helicobacter* DNAs were non-pylori *Helicobacter* species (Bohr et al., 2004). By using a *Helicobacteraceae* family specific PCR, a significantly higher presence of members of *Helicobacteraceae* were found in intestinal biopsies and fecal samples from children with CD (Zhang et al., 2006; Man et al., 2008; Kaakoush et al., 2010). Further identification of the PCR amplified DNA revealed that most of the detected members of *Helicobacteraceae* were enteric *Helicobacter* species (non-pylori *Helicobacter* species that colonize the intestinal tract). In intestinal biopsies and luminal washings collected from adult patients with IBD and controls, a study from UK detected non-pylori *Helicobactor* by PCR in 2.9% patients with IBD and 8.1% controls (Basset et al., 2004). Studies of detection of enteric *Helicobacter* species in IBD were mainly conducted by PCR. The lack of isolated enteric *Helicobacter* species from patients with IBD has prevented the study of pathogenic mechanisms of these bacterial species.

### Campylobacter concisus

*C. concisus* is an oral bacterium and some strains can also colonize the intestinal tract (Zhang, 2015; Liu et al., 2018b). The association between *C. concisus* and pediatric CD was first reported using PCR detection of *C. concisus* 16S rRNA gene in intestinal biopsies and bacterial isolation (Zhang et al., 2009). The association between pediatric CD and *C. concisus* was then demonstrated using fecal samples (Man et al., 2010b). Later studies also reported the association between *C. concisus* and IBD, including both CD and UC, in the adult population (Mahendran et al., 2011; Mukhopadhya et al., 2011; Kirk et al., 2016). Genome analysis showed that *C. concisus* contains two genomospecies, and *C. concisus* strains isolated from intestinal tract of patients with IBD were predominately genomospecies 2 (Chung et al., 2016; Wang et al., 2017; Cornelius et al., 2021). Analysis of genomes of *C. concisus* strains isolated from patients with IBD and healthy controls also identified molecular markers for strains that were associated with severe CD and UC, these markers were from genomospecies 2 *C. concisus* strains (Liu et al., 2018a; Liu F. et al., 2020). Several studies also examined the pathogenic mechanisms that may contribute to the pathogenesis of IBD.

*C. concisus* damages the intestinal epithelial barrier. The bacterium induced apoptosis in human intestinal epithelial HT-29 cells following 48 h incubation and the expression of barrier-forming tight junction protein claudin-5 reduced to 66% (Nielsen et al., 2011). Tight junction protein redistribution caused by *C. concisus* was also observed (Man et al., 2010a; Nielsen et al., 2011). *C. concisus* strain adhered to and some strains were invasive to intestinal epithelial cells (Man et al., 2010a; Ismail et al., 2012). Some *C. concisus* strains have acquired prophages that contain the *zot* gene (Liu et al., 2016). The *C. concisus* Zot protein caused cell death in human intestinal epithelial Caco-2 cells (Mahendran et al., 2016).

*C. concisus* enhances the responses of intestinal epithelial cells and macrophages to commensal bacteria. Preincubation of THP-1 macrophage-like cells and HT-29 cells with *C. concisus* Zot protein significantly enhanced the production of TNF-α by these cells in response to *E. coli* (Mahendran et al., 2016). Incubation of HT-29 cells with *C. concisus* upregulated the expression of TLR4 and MD-2, which primarily recognize LPS (Ismail et al., 2013). LPS is a core component of the outer membrane of Gram-negative bacteria. A low level of expression of TLRs is a mechanism of maintain the tolerance to gut bacteria (Santaolalla and Abreu, 2012). Increased expression of TLR4 and MD-2 on intestinal epithelial cells may promote these cells to respond to commensal gut microbes that they usually do not respond under homeostasis, which causes intestinal inflammation. *C. concisus* has a spiral to curved shape, a morphology that better equips bacterial species with abilities to travel in mucus and get close to epithelium.

*C. concisus* induces intestinal inflammation. *C. concisus* induced the production of IL-8 in intestinal epithelial cells (Man et al., 2010a; Ismail et al., 2013), and the production of IL-8 and TNF-α in human macrophages (Man et al., 2010a; Mahendran et al., 2016).

*C. concisus* also has the potential to affect the adaptive immune system. *C. concisus* upregulated epithelial expression of the immune checkpoint protein PD-L1 in interferon (IFN)-γ sensitized HT-29 cells, suggesting that while *C. concisus* induces innate inflammatory responses, it has the potential to inhibit the function of T cells (Lee et al., 2021). However, the upregulated expression of PD-L1 was only examined at an mRNA level. Whether the upregulated PD-L1 has functional bioactivity at a protein level is not yet known.

An individual’s gastrointestinal environment has an impact on the enteric pathogenicity of *C. concisus*. The growth of *C. concisus* is highly dependent on the presence of H_2_ (Lee et al., 2014). In the human intestinal tract, the level of H_2_ gas generated by bacterial species is greatly affected by diet and gut bacterial composition, which may play a role in determining *C. concisus* intestinal colonization (Strocchi and Levitt, 1992).

### Fusobacterium nucleatum

*F. nucleatum* is a Gram-negative anaerobe that colonizes the human oral cavity and intestinal tract. A study from Canada in 2011 reported a significantly higher isolation of *F. nucleatum* from intestinal biopsies of patients with IBD as compared with healthy controls (Strauss et al., 2011). Recently, *F. nucleatum* was reported to be abundant in intestinal tissues of patients with UC and IBD, and the abundance was associated with disease severity (Liu H. et al., 2020; Su et al., 2020).

The pathogenic mechanisms of *F. nucleatum* relating to IBD include damaging intestinal barrier and promoting inflammation. *F. nucleatum* strains isolated from inflamed biopsies of patients with IBD were more invasive to Caco-2 cells (Strauss et al., 2011). In mice with dextran sodium sulfate (DSS)-induced colitis, *F. nucleatum* promoted intestinal inflammation (Liu H. et al., 2020; Su et al., 2020). *F. nucleatum* damaged intestinal epithelial barrier by inducing autophagic epithelial cell death, affecting the expression and distribution of zonula occludens-1 (ZO-1) and occludin and skewing proinflammatory M1 macrophage (Liu L. et al., 2019; Liu H. et al., 2020; Su et al., 2020). In mice with DSS-induced colitis, *F. nucleatum* induced Th1 and Th17 subset T cell differentiations, promoted the secretion of proinflammatory cytokines such as TNF-α, IFN-γ, IL-1β, IL-6, and IL-17 (Liu L. et al., 2019).

*F. nucleatum* is also associated with colorectal cancer (Lee et al., 2019). Using colorectal cancer cell lines (HCT116, DLD1, SW480, and HT-29) and xenograft mice, it was shown that *F. nucleatum* promotes colorectal cancer cell growth by modulating E-cadherin and promotes cancer cell proliferation via FadA adhesin, activating TLR4 signaling to nuclear factor-kB (Rubinstein et al., 2013; Yang et al., 2017).

### Changed Gut Microbiota in Inflammatory Bowel Disease

In recent years, numerous studies have compared the gut microbiota in patients with IBD and controls. These studies were recently reviewed by Pittayanon et al. (2020) in a systematic review published in Gastroenterology. Pittayanon et al. (2020) reviewed 48 such studies and found the following: (1) The diversity of microbiota was either decreased or not different in patients with IBD vs. controls. (2) For patients with CD, three studies reported a decrease of *Christensenellaceae* and *Coriobacteriaceae* as compared to controls. *Faecalibacterium prausnitzii* was reported to decrease in 6 of 11 studies. Two studies each of *Actinomyces*, *Veillonella* and *E. coli* revealed an increase of these organisms. (3) For patients with UC, *Eubacterium rectale* and *Akkermansia* were decreased in three studies, *E. coli* was increased in four of nine studies. (4) Pittayanon et al. (2020) stated that they cannot make conclusions due to inconsistent results and methods among studies.

## Discussion

As stated above, several bacterial species were found to be positively associated with IBD, suggesting that they may play a role in the development of IBD.

IBD is not a single disease. The diagnosis of IBD is based on histopathology and inflammatory markers rather than microbes that trigger the development of the inflammatory conditions. Immune responses to different microbes may result in a similar histopathology. It is therefore not surprising that multiple bacterial species were found to be associated with IBD. Bacterial species that are associated with IBD were from different sources, including the oral cavity, intestinal tract, animal, or environment. *C. concisus* and *F. nucleatum* are oral bacterial species. The oral cavity is a constantly available source providing these organisms to the intestinal tract. In individuals with high numbers of *C. concisus* or *F. nucleatum* being translocated to the intestinal tract or with an intestinal microenvironment that is suitable for the growth of these organisms, *C. concisus* or *F. nucleatum* may reach sufficient numbers to cause pathogenic effects in the intestinal tract.

**FIGURE 1:**
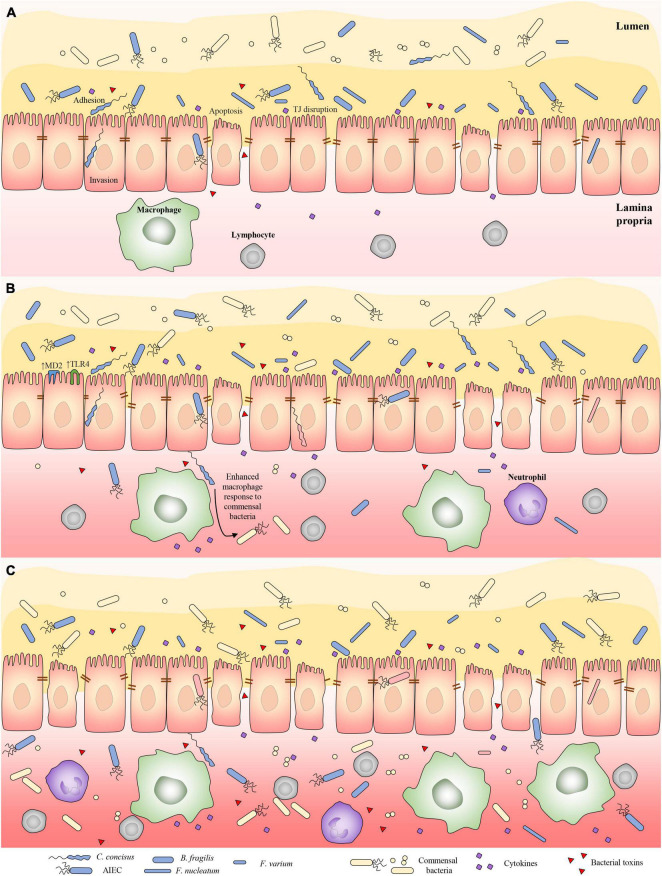
Three-stage pathogenesis model illustrating the role of initiating bacterial species and gut commensal bacterial species in contributing to the development of IBD. (A) In stage one, bacterial species that have abilities overcoming the mucosal defense cause damage to intestinal barrier and induce intestinal inflammation. Proinflammatory cytokines induced by these bacterial species further damage the intestinal barrier. Most of IBD-associated bacterial species in Table 1 belong to IBD-initiating bacterial species. Other enteric pathogens may also increase the risk of developing IBD if they cause prolonged infections. (B) In stage two, prolonged intestinal barrier damage caused by the IBD-initiating bacterial species and their abilities to enhance the response of mucosal immune system to gut commensal bacterial species lead to the breakdown of the homeostasis between the mucosal immune system and the gut microbiota, thus the gut commensal bacteria become part of the microbes that induce inflammation in the pathogenesis of IBD. (C) In the third stage of IBD pathogenesis, the gut commensal microbes are the main drivers of the intestinal inflammation due to their large amount, via both innate and adaptive immune responses. Inflammatory conditions are a changed ecosystem to gut microbes. Anaerobes are likely to disappear under an inflammatory environment due to their reduced ability to deal with oxidative stress as compared to facultative microbes. TJ, tight junction.

We propose a three-stage pathogenesis model to illustrate the roles of IBD-associated bacterial species and gut commensal bacterial species in the development of IBD (Figure 1). In the first stage of IBD development, the main players in inducing inflammation are the initiating microbes, which have the ability to overcome the mucosal defense system. Despite possessing different virulence factors, *C. concisus*, *F. nucleatum, B. fragilis*, *F. varium*, AIEC, *C. difficile* and many enteric pathogens share common pathogenic effects. They damage the intestinal epithelial cells and cause intestinal inflammation. Proinflammatory cytokines induced by these bacteria further damage the intestinal barrier (Liu F. et al., 2019). Most of the IBD-associated bacterial species are likely the initiating microbes contributing to stage 1 of IBD pathogenesis. Other enteric pathogens may also increase the risk of developing IBD if they cause prolonged infections.

In the second stage of IBD development, both the initiating microbes and some gut commensal bacteria contribute to the pathogenesis. The continuous supply of *C. concisus* and *F. nucleatum* to the intestinal tract from the oral cavity and the fact that *B. fragilis*, AIEC and *C. difficile* are residing enteric microbes make it difficult to eliminate these bacteria by immune responses. These microbes are therefore likely to cause prolonged intestinal barrier damage and inflammation, which leads to a long-term increased passage of the gut commensal microbes and their products to intestinal tissues. Some of the initiating bacterial species also promote the responses of mucosal immune system to commensal bacteria. For example, *C. concisus* enhances the responses of macrophages to commensal bacterium *E. coli* and increases the expression of microbial recognition receptors on intestinal epithelial cells (Ismail et al., 2013; Mahendran et al., 2016). These effects eventually lead to the breakdown of the homeostasis between the mucosal immune system and the gut microbiota, thus some of the gut commensal bacteria become part of the microbes that induce inflammation in the pathogenesis of IBD (Figure 1).

In the third stage of IBD pathogenesis, the gut commensal microbes become the main drivers of the intestinal inflammation due to their large amount, via both innate and adaptive immune responses. Upon intestinal barrier damage, individual’s gut microbial communities determine the severity of intestinal inflammation, which has been clearly demonstrated using DSS colitis model (Roy et al., 2017). Bacterial species have different abilities in resistant inflammatory mediators released by the host. At this stage, some initiator microbes that have less abilities in dealing with oxygen stress such as *C. concisus* may not be able to survive long in an environment with severe inflammation (Yeow et al., 2020). The others such as *E. coli* thrive in the inflamed gut, due to its abilities to resist anti-bacterial molecules released by the host (Singh et al., 2015).

*M. avium* species is wildly present in the environment and in the gastrointestinal tract of animals. It usually only causes diseases in individuals with severely damaged immune system such as HIV patients with extremely low CD4^+^ T cells, underlying lung disease or sometimes in young children, and chronic obstructive pulmonary disease (Whiley et al., 2012). IBD most often occurs in young adults, which is a period with relatively strong immunity in the human life (Johnston and Logan, 2008). This disease pattern does not seem to support an initiating role of MAP in the pathogenesis of human IBD.

Numerous studies have examined the gut microbiota in patients IBD in the last decade. Most of these studies found reduced bacterial diversities and dysbiosis as compared to non-IBD controls. However, bacterial species contributing to the dysbiosis reported in different studies varied greatly. A recent systematic review analyzing 48 of such studies stated that they cannot draw conclusions due to inconsistent results and methods among studies (Pittayanon et al., 2020). Given the impacts of diet, inflammation, drugs, genetics, and many other factors on the growth of different bacterial species, the cross-feeding and inhibition between bacterial species in the gut microbiota, it is indeed a great challenge to find reliable relative gut bacterial abundance as markers to indicate IBD, which is not a single disease. The common feature of this group of disease is inflammation, which is caused by the same or different microbes. Inflammatory conditions are a changed ecosystem to gut microbes. The bacterial species in the human intestinal tract are largely anaerobes and facultative anaerobes. Anaerobes are likely to disappear under an inflammatory environment due to their limited ability to deal with oxidative stress as compared to facultative microbes. A recent study using a multiomics approach analyzing gut microbiota in patients with IBD and controls with 24 months follow up indeed has demonstrated this (Lloyd-Price et al., 2019). As mentioned above, with a damaged intestinal barrier caused by the IBD-associated bacterial species and the inflammatory cytokines, gut commensal bacterial species, can contribute to, and even become the main player in the pathogenesis of IBD due to their large amount at the third stage of IBD pathogenesis. Studies of gut microbiota predominantly used universal primers targeting 16S rRNA gene, which favors the amplification of dominant bacterial species and can only identify some bacteria to a species level. The minority bacterial species including some of the IBD-initiating bacterial species may not be detected using this approach. Indeed, the IBD-associated bacterial species listed in Table 1 were detected by specific PCR and bacterial isolation.

Current treatment for IBD mainly include anti-inflammatory drugs, immune suppressive drugs and monoclonal antibodies targeting TNF-α, IL-12, and integrin or immunosuppressive drugs. These drugs in general target the host immune response, except that immunosuppressive azathioprine and mercaptopurine have inhibitory effects to some bacterial species of the gastrointestinal tract including the IBD-associated *C. concisus* (Liu et al., 2017). For clinically diagnosed patients with IBD, most of them would be at the second or third stages of the proposed pathogenesis model. Given that microbes in the gastrointestinal tract, including both the gut commensal and the initiating microbes, are the drivers of the inflammation at these stages. It makes sense that strategies targeting these initiating microbes and commensal microbes should be included for the management of IBD. We recommend some anti-microbial strategies in additional to the currently used IBD therapies. The first strategy we suggest is to reduce the load of IBD-associated bacterial species in the oral cavity, which can in turn reduce the translocation of these bacteria to the intestinal tract. This can be achieved by physical or chemical procedures targeting bacteria in the oral cavity. The second strategy is to reduce interactions of gut commensal bacteria with the intestinal epithelium and cells of the mucosal immune system. Under chronic inflammatory conditions in the second and third stages of IBD pathogenesis, the relationship between commensal gut bacterial species and the host has changed. These microbes are not commensal to the host anymore but pathogens driving inflammation. Reduction of gut microbes and elimination of triggering microbes would be the most effective way to end the immune responses caused by them and restore the intestinal barrier. This may be best achieved by enema or other locally delivered antimicrobial agents targeting the lesion areas.

## Author Contributions

LZ wrote the first version of the manuscript. FL generated the figure. FL, JX, SL, LL, and SR provided critical feedback and helped with editing the manuscript. All authors contributed to the article and approved the submitted version.

## Conflict of Interest

The authors declare that the research was conducted in the absence of any commercial or financial relationships that could be construed as a potential conflict of interest.

## Publisher’s Note

All claims expressed in this article are solely those of the authors and do not necessarily represent those of their affiliated organizations, or those of the publisher, the editors and the reviewers. Any product that may be evaluated in this article, or claim that may be made by its manufacturer, is not guaranteed or endorsed by the publisher.
